# Predictive value of PD-L1 expression to the efficacy of immune checkpoint inhibitors in advanced triple-negative breast cancer: A systematic review and meta-analysis

**DOI:** 10.3389/fphar.2022.1004821

**Published:** 2022-12-02

**Authors:** Yingjie Qi, Xin Yan, Chao Wang, Hui Cao, Guangxuan Liu

**Affiliations:** ^1^ Department of Pharmacy, Cancer Hospital of China Medical University, Liaoning Cancer Hospital and Institute, Shenyang, China; ^2^ School of Life Science and Biopharmaceutics, Shenyang Pharmaceutical University, Shenyang, China; ^3^ Institute of Drug Control, Liaoning Inspection, Examination and Certification Centre, Shenyang, China; ^4^ Department of Breast Medicine, Cancer Hospital of China Medical University, Liaoning Cancer Hospital and Institute, Shenyang, China

**Keywords:** triple-negative breast cance, immune checkpoint inhibitors, PD-L1 expression, efficacy, meta-analysis

## Abstract

**Background:** Immune checkpoint inhibitors (ICIs) have been an emerging treatment strategy for advanced triple-negative breast cancer (TNBC). Some studies have shown that high expression of programmed death-ligand 1 (PD-L1) can achieve a better response of clinical efficacy. However, the efficacy of ICIs in advanced TNBC remains controversial. In this meta-analysis, we evaluated the correlation of PD-L1 expression with the efficacy of ICIs in patients with advanced TNBC.

**Methods:** We conducted a systematic search using four databases until March 2022 to obtain eligible randomized controlled trials (RCTs). The quality of the studies was assessed by the Cochrane risk of bias tool. Hazard ratio (HR) was extracted to evaluate the relationship between PD-L1 expression and progression-free survival (PFS) or overall survival (OS) in patients with advanced TNBC.

**Results:** Five randomized controlled clinical trials (RCTs) with 3104 patients were included in this meta-analysis. The results demonstrated that ICIs could significantly improve the OS (HR 0.77, 95% CI 0.60–0.98, *p* = 0.03) in PD-L1 positive TNBC group. In the subgroup analysis, longer OS was observed (HR: 0.70, 95% CI: 0.60–0.82, *p* = 0.00001) in PD-L1 positive TNBC patients receiving ICIs alone or ICIs combined with nab-paclitaxel. In terms of PFS, PFS was significantly improved (HR: 0.68, 95% CI: 0.58–0.79, *p* < 0.00001) in PD-L1 positive patients receiving first-line ICIs and chemotherapy compared to those with ICIs alone. No significant improvement was observed for OS or PFS in PD-L1 negative group.

**Conclusion:** Our study indicated significant improvement for OS in advanced TNBC with ICIs therapy in the PD-L1 positive status, and ICIs alone or ICIs combined with nab-paclitaxel might be a excellent choice in terms of OS. Although PFS has no significant benefit in PD-L1 positive patients, the subgroup analysis showed that ICIs combined with chemotherapy could achieve the PFS benefit in the first-line treatment. However, further clinical studies are needed to validate our conclusions due to limited relevant research.

## Introduction

Breast cancer has the highest incidence of all malignant tumors among women worldwide (Zhang et al., 2021). Triple-negative breast cancer (TNBC) accounts for approximately 15–20% of all breast cancers. Its pathological features are negative for estrogen receptor (ER), progesterone receptor (PR), and human epidermal growth factor receptor 2 (HER-2), characterized by an early age of onset, strong invasiveness and high recurrence and metastasis rate (Li et al., 2016; Sharma et al., 2018). The clinical prognosis of TNBC patients is poor due to the lack of effective targets for endocrine therapy and targeted therapy. The 5-year survival rate of patients with advanced TNBC is less than 15% (Bagegni et al., 2019; Berger et al., 2021). Immunotherapy provides a new treatment strategy for the patients. Pathologically, TNBC has relatively abundant tumor-infiltrating lymphocytes and high expression level of programmed death-ligand 1 (PD-L1), providing a suitable immune microenvironment and target basis for the application of immune checkpoint inhibitors (ICIs) (Disis et al., 2015; García-Teijido et al., 2016; Huang et al., 2021). Clinical trials such as KEYNOTE-012, KEYNOTE-086, and IMpassion 031 have confirmed that the immune checkpoint programmed cell death-1 (PD-1)/PD-L1 inhibitors are effective treatment options for TNBC (Seiwert et al., 2016; Adams et al., 2019; Mittendorf et al., 2020). However, only a small proportion of patients showed a long-term sustained response (Keenan et al., 2020). Therefore, there is an urgent need for reliable biomarkers of immune therapy to screen out the optimal beneficiary population clinically (Emens et al., 2021). PD-L1 expression level is currently the most important and controversial predictor of immunotherapy efficacy. IMpassion130 trial indicated that atezolizumab in combination with nab-paclitaxel significantly increased the overall survival (OS) and the progression-free survival (PFS) in PD-L1 positive patients with metastatic TNBC (Schmid et al., 2020), but, KEYNOTE-119 trial demonstrated that pembrolizumab did not significantly improved the OS in PD-L1 positive patients with previously treated metastatic TNBC *versus* chemotherapy (Winer et al., 2021).

In recent years, several studies have investigated the association between PD-L1 and immunotherapy clinical outcomes in TNBC, but most of them focused on the early stage of TNBC. Two meta-analysis studied the prognostic role of PD-L1 in advanced or metastatic TNBC (Ji et al., 2021; Latif et al., 2022), they conducted the subgroup analysis stratifying the status of PD-L1 by including two studies, and the results suggested that better PFS or OS could be found in PD-L1 positive group. Notably, these meta-analyses were short of the latest relevant clinical trials and further subgroup analysis such as ICIs alone or ICIs combination with other chemotherapeutic drugs. In this study, we summarized recent and relevant clinical trials and conducted a meta-analysis of randomized controlled trials (RCTs) to assess the relationship between PD-L1 expression and the efficacy of ICIs in locally advanced or metastatic TNBC.

## Materials and methods

### Literature search

PubMed, Embase, Cochrane Library, and Web of Science databases were searched to collect and select clinical studies on PD-L1 expression and PD-1/PD-L1 inhibitors for the treatment of TNBC published before March 2022 and the following keywords were used: “immune checkpoint inhibitor” or “PD-L1” or “PD-1” or “durvalumab” or “pembrolizumab” or “atezolizumab” or “nivolumab” or “avelumab” or “ipilimumab” or “tremelimumab” and “triple-negative breast cancer”. We also manually screened the relevant studies in the references to retrieve other eligible literatures.

### Study selection

Eligible studies met the following inclusion criteria:1) studies should be randomized controlled trials (RCTs) in patients with locally advanced or metastatic TNBC in stage III-IV; 2) the expression of PD-L1 in patients was detected by immunohistochemistry (IHC); 3) study evaluated the efficacy of ICIs or ICIs combined with chemotherapy; 4) studies directly provided hazard ratio (HR) of OS or PFS and 95% confidence intervals (95% CIs) or they could be calculated indirectly in patients with different PD-L1 expression. 5) The language of the publication was English. Articles were excluded according to the following criteria: 1) patients with early stage TNBC; 2) conference abstracts, reviews, case reports, or non-RCTs; 3) the latest and most complete study was chosen between the same studies in different periods, others were excluded.

## Data extraction

The studies were independently evaluated and extracted according to the inclusion and exclusion criteria by two reviewers, and the disagreements between them were resolved by discussion. The following data from the eligible study were retrieved: name of the trial, year of publication, study phase, study population, therapeutic regimen, rate of PD-L1 positive expression, cutoff value, HRs, and 95% CI of OS and PFS, if available. The latest and most complete study was chosen between the same studies in different periods.

### Quality assessment

The quality of the included studies was assessed using the assessment criteria provided by the Cochrane Collaboration bias assessment tool version 5.4. The criteria were based on seven aspects: parameters of details of random sequence generation, allocation concealment, blinding for participants and personnel, blinding for outcome assessment, incomplete outcome data and selective reporting. In addition, the risk of bias was divided into three levels: low risk, high risk and unclear.

### Statistical analysis

All data were statistically analyzed using the Review Manager software (RevMan, version 5.3 for windows; Cochrane Collaboration, Oxford, United Kingdom) and STATA version 16.0 software. HR and 95% CIs of OS and PFS were directly extracted. HR < 1 indicated that the survival outcomes were better in immunotherapy group compared with chemotherapy group. HR > 1 indicated the opposite. The pooled HRs were considered statistically significant if *p* < 0.05 (two-sided). The heterogeneity between studies was assessed using Cochran Q-test and *I* ^*2*^ statistics. A *p* ≤ 0.10 or *I* ^*2*^ ≥ 50% indicated the existence of heterogeneity among studies, and a random-effects model was adopted; A *p* > 0.10 or *I* ^*2*^ < 50% indicated the absence of heterogeneity among studies, and a fixed-effects model was used.

## Results

### Study selection and characteristics

**FIGURE 1:**
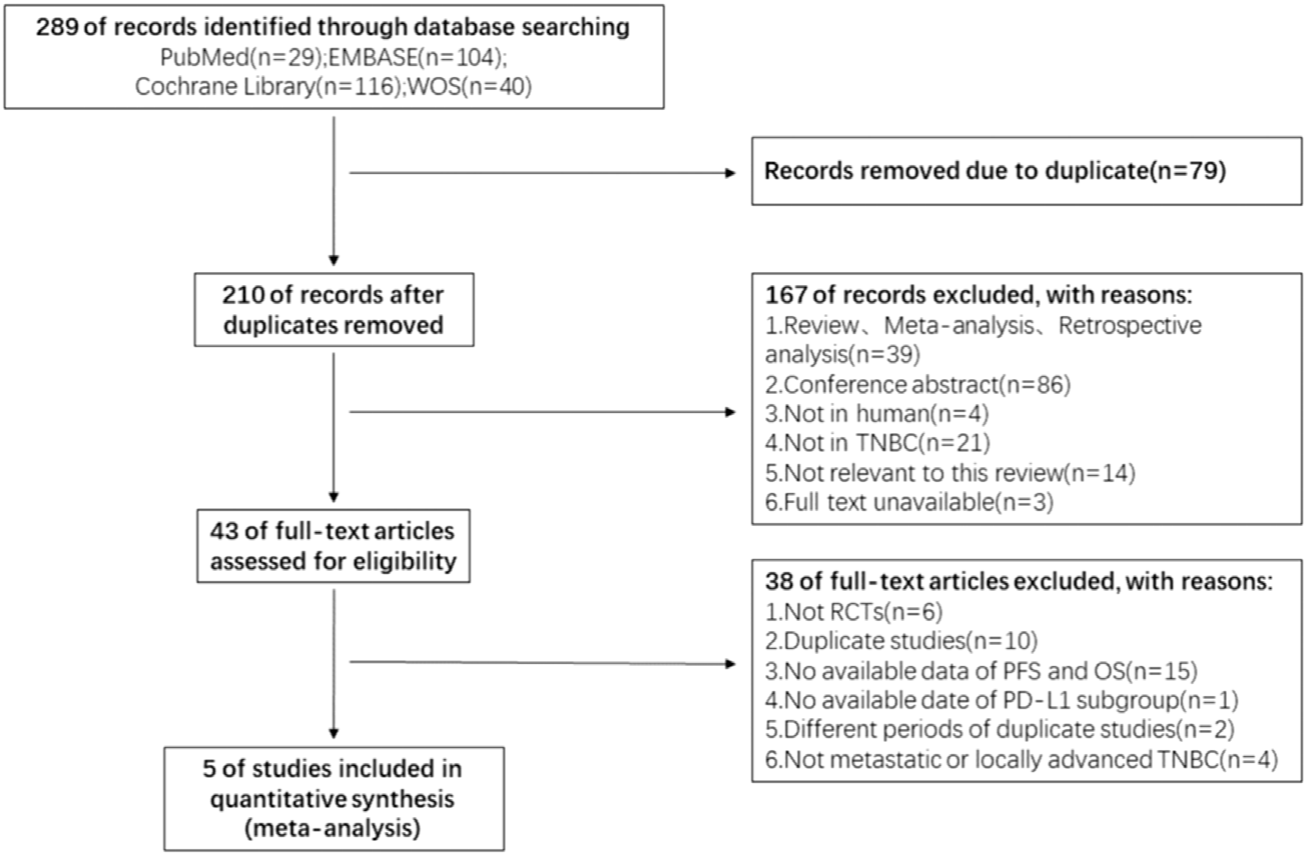
Flow chart of the literature search and study selection.

A total of 289 related literature were retrieved and 210 were retained after excluding 79 repeated studies. The authors browsed the titles and abstracts, 46 papers were screened by excluding reviews, conference abstracts, and non-anthropological studies. According to the inclusion criteria of this study, five eligible RCTs [KEYNOTE-119 (Winer et al., 2021), KEYNOTE-355 (Cortes et al., 2020), IMpassion 130 (Schmid et al., 2020), IMpassion131 (Miles et al., 2021), SAFIR02-BREAST IMMUNO(Bachelot et al., 2021)] studies were finally included after reading the full text. A flow chart of the study selection is presented in Figure 1.

**FIGURE 2:**
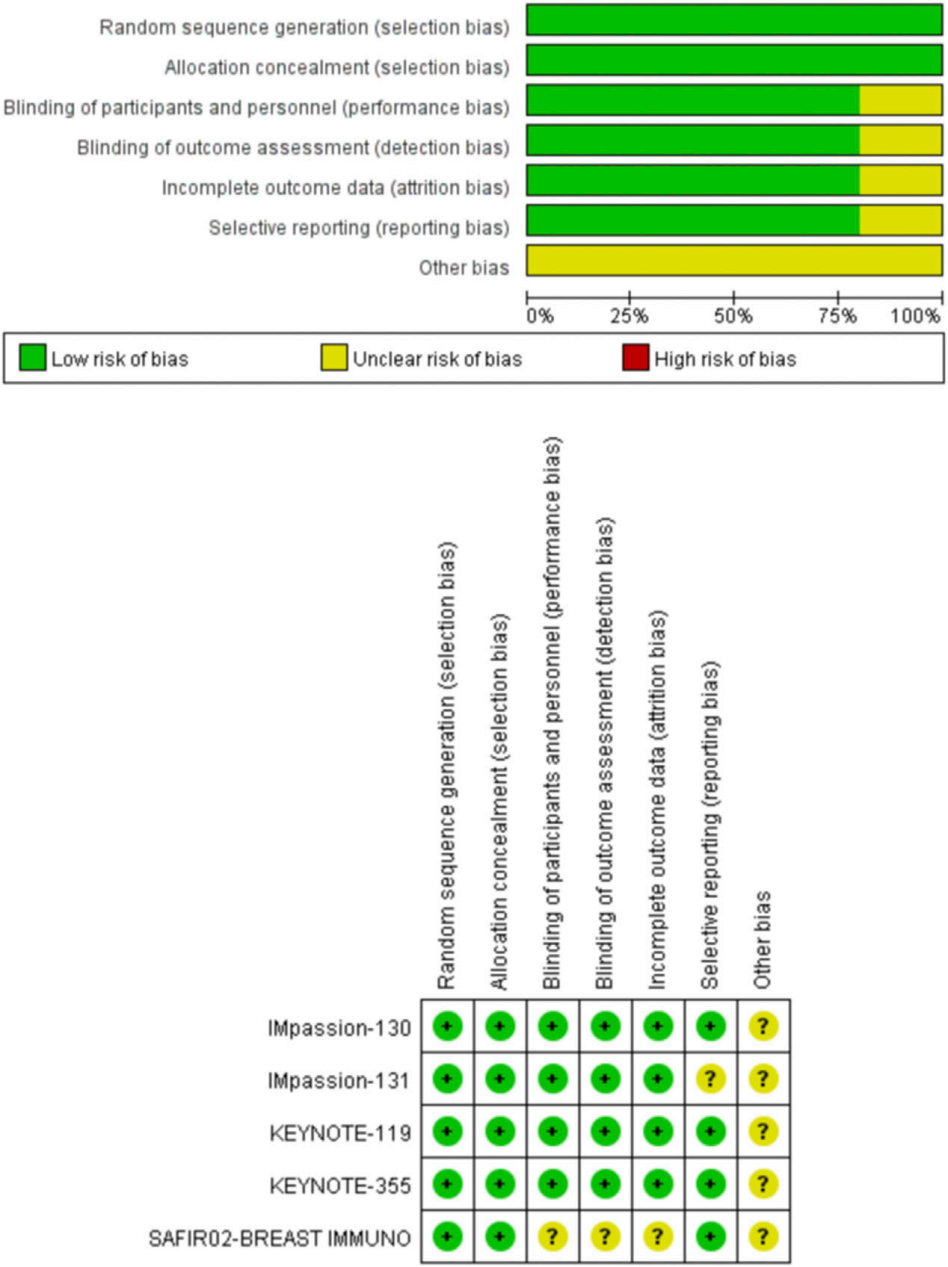
Quality assessment for risk of bias for the included randomized controlled trials.

A total of 3104 patients with TNBC were enrolled, of whom 1734 were PD-L1 positive. The five RCTs were published between 2019 and 2021, which included four phase III studies and one phase II study. The ICIs involved were atezolizumab, pembrolizumab, and durvalumab. In addition, three studies compared the efficacy of ICIs combined with chemotherapy *versus* chemotherapy and two studies compared the efficacy of ICIs alone *versus* chemotherapy. Table 1 presents the characteristics of the five RCTs. The results of the quality evaluation are shown in Figure 2.

**TABLE 1:** Characteristics of the included studies in the meta-analysis.

Study	Year	Phase	Population	Treatment characteristics	Patient with pd-l1positive/total (%)	Cutoff value	Antibody	PFS HR (95% CI)	OS HR (95% CI)
IMpassion 131	2021	III	Stage IVmTNBC	Atezolizumab + p	292 (44.9)	IC ≥ 1%	SP142	0.82 (0.60, 1.12)	1.55 (0.86, 2.79)
KEYNOTE-355	2020	III	Stage IV mTNBC	Pembrolizumab + Nab-P/P/GC	323 (38.1)	CPS≥10	22C3pharmDx	0.65 (0.49, 0.86)	0.72 (0.55, 0.95)
IMpassion 130	2020	III	StageIII-IV aTNBC/mTNBC	Atezolizumab + Nab-P	369 (40.9)	IC ≥ 1%	SP142	0.63 (0.50, 0.79)	0.67 (0.53, 0.85)
SAFIR02-BREAST IMMUNO	2021	II	Stage IV mTNBC	Durvalumab	32 (39.0)	IC ≥ 1%	SP142	NR	0.37 (0.12, 1.14)
KEYNOTE-119	2021	III	Stage IV mTNBC	Pembrolizumab	194 (31.2)	CPS≥10	22C3pharmDx	1.14 (0.82, 1.59)	0.78 (0.57, 1.06)

mTNBC, metastatic triple-negative breast cancer; aTNBC, advanced triple-negative breast cancer; *p*, paclitaxel; Nab-P, nab-paclitaxel; GC, gemcitabine–carboplatin; CPS, combined positive score, the PD-L1 CPS, was defined as number of PD-L1–positive cells (tumour cells, lymphocytes, and macrophages) divided by total number of tumour cells × 100; IC, tumor-infiltrating immune cells, it was defined as 1% or higher programmed death-ligand 1 (PD-L1)–expressing tumor-infiltrating immune cells; PFS, progression-free survival; OS, overall survival; HR, hazard ratio; 95% CI, 95% confidence interval; NR, not reported.

### Association of PD-L1 expression with PFS

**FIGURE 3:**
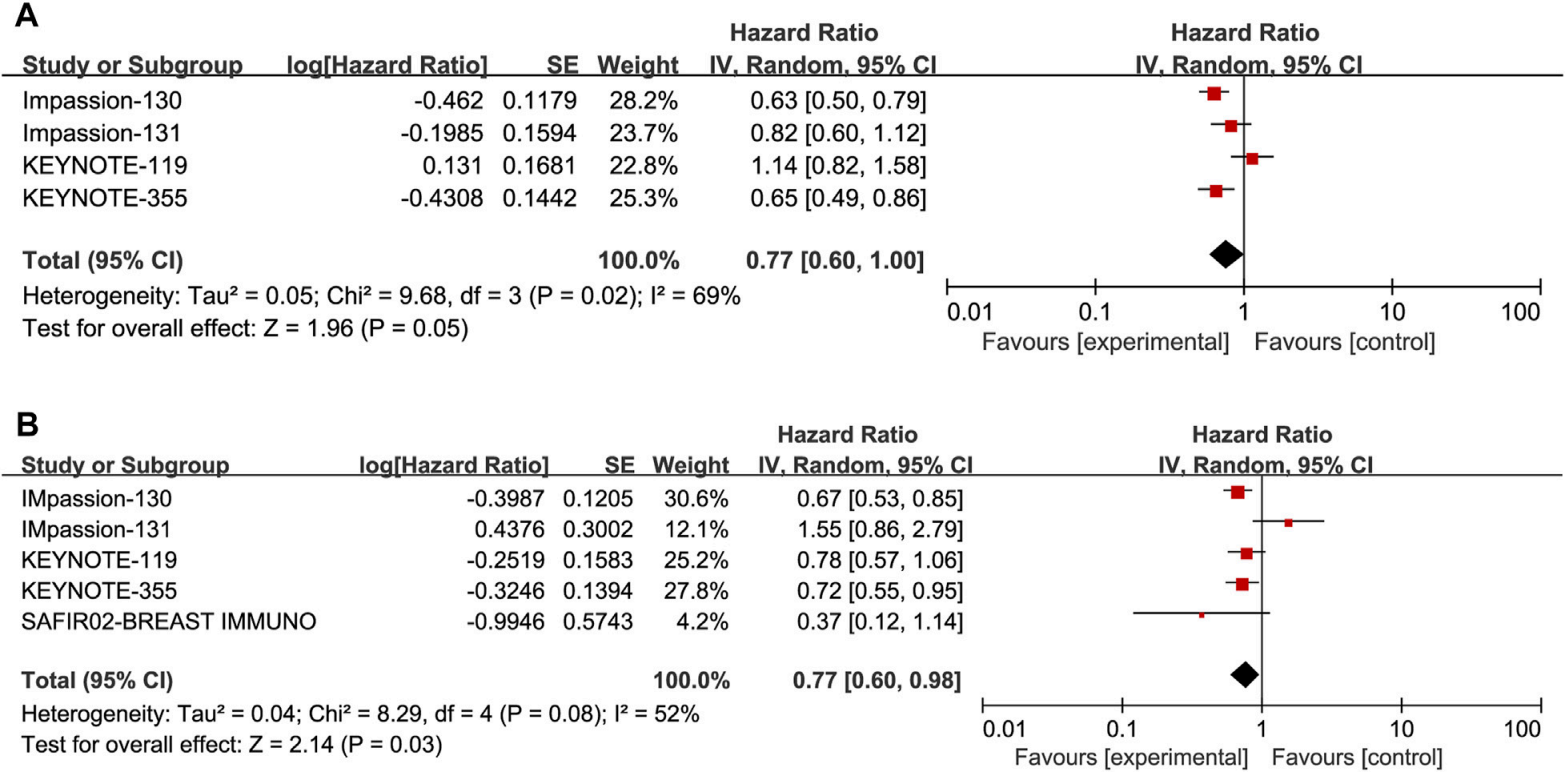
Forest plots of meta-analyses between ICIs combination with chemotherapy or not vs chemotherapy in PD-L1 positive TNBC **(A)** for progression-free survival and **(B)** for overall survival.

**FIGURE 4:**
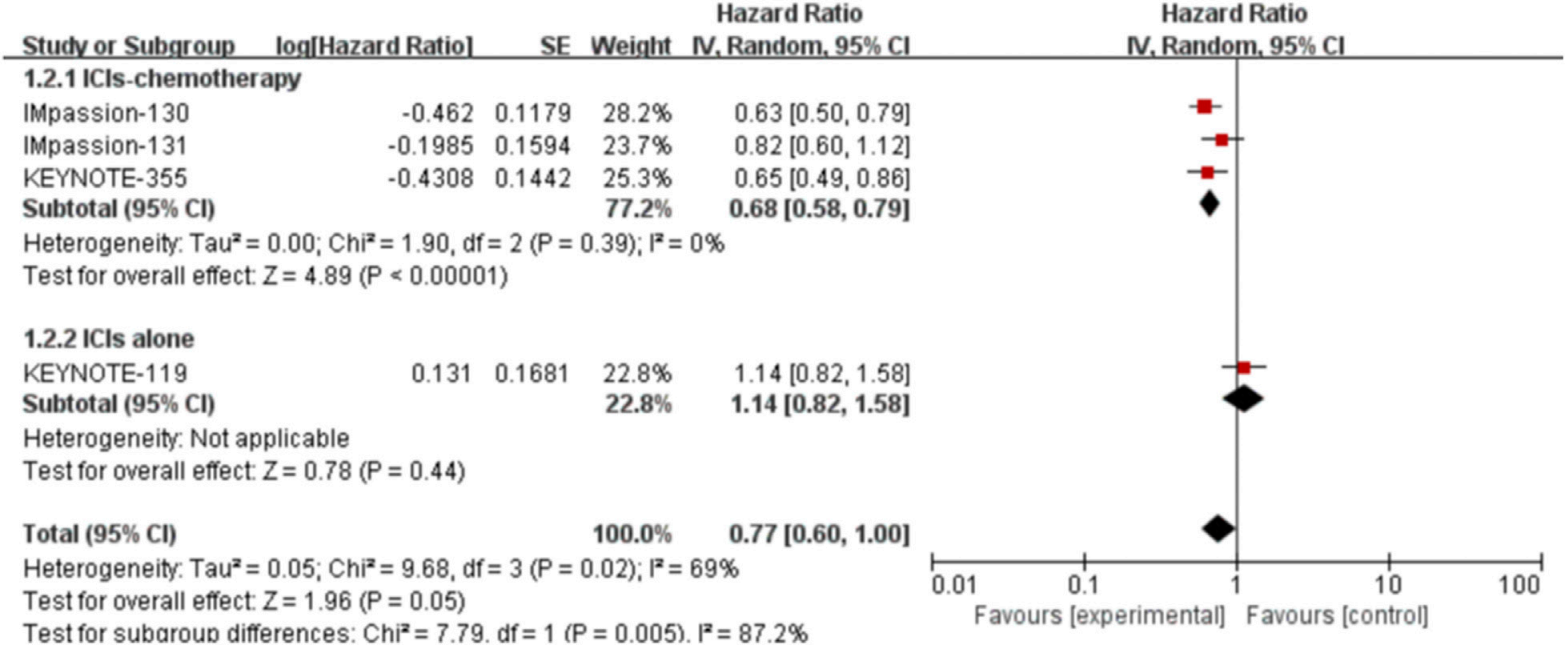
Subgroup analysis of the effect of ICIs combined with chemotherapy or not on PFSin PD-L1 positive TNBC patients.

Four studies, including 3022 patients, reported the data of PFS with 1702 patients in the PD-L1 positive group and 1320 patients in the PD-L1 negative group. In the PD-L1 positive group, we adopted a random-effects model for analysis due to considerable heterogeneity (*I* ^*2*^ = 69%, *p* = 0.02). No statistically significant result was observed in the correlation between the efficacy of ICIs and PFS in the PD-L1 positive locally advanced or metastatic TNBC (HR: 0.77, 95% CI: 0.60–1.00, *p* = 0.05; Figure 3). The subgroup analysis was performed by stratifying the treatment project and treatment line to explore the sources of heterogeneity among studies. The subgroup analysis results showed that patients could achieve increased PFS in the PD-L1 positive group if they received ICIs combined with chemotherapy in first-line treatment (HR: 0.68, 95% CI: 0.58–0.79, *p* < 0.00001; Figure 4).

In the PD-L1 negative group, two studies reported relevant data. We used a fixed-effects model for analysis due to the absence of heterogeneity (*I* ^*2*^ = 0%, *p* = 0.39). The result also indicated no significant improvement in PFS in patients who received ICIs (HR: 0.93, 95% CI: 0.81–1.08, *p* = 0.35).

### Association of PD-L1 expression with OS

**FIGURE 5:**
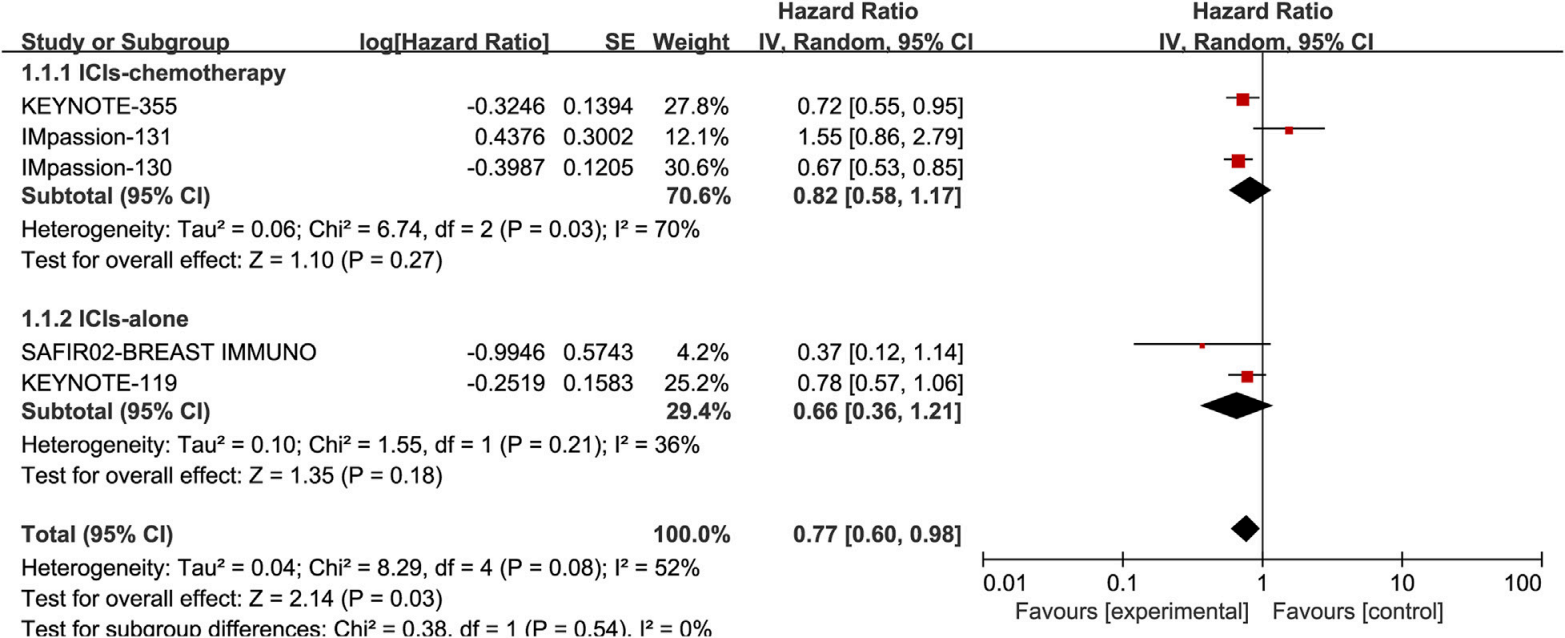
Subgroup analysis of the effect of ICIs combined with chemotherapy or not on OS in PD-L1 positive TNBC patients.

**FIGURE 6:**
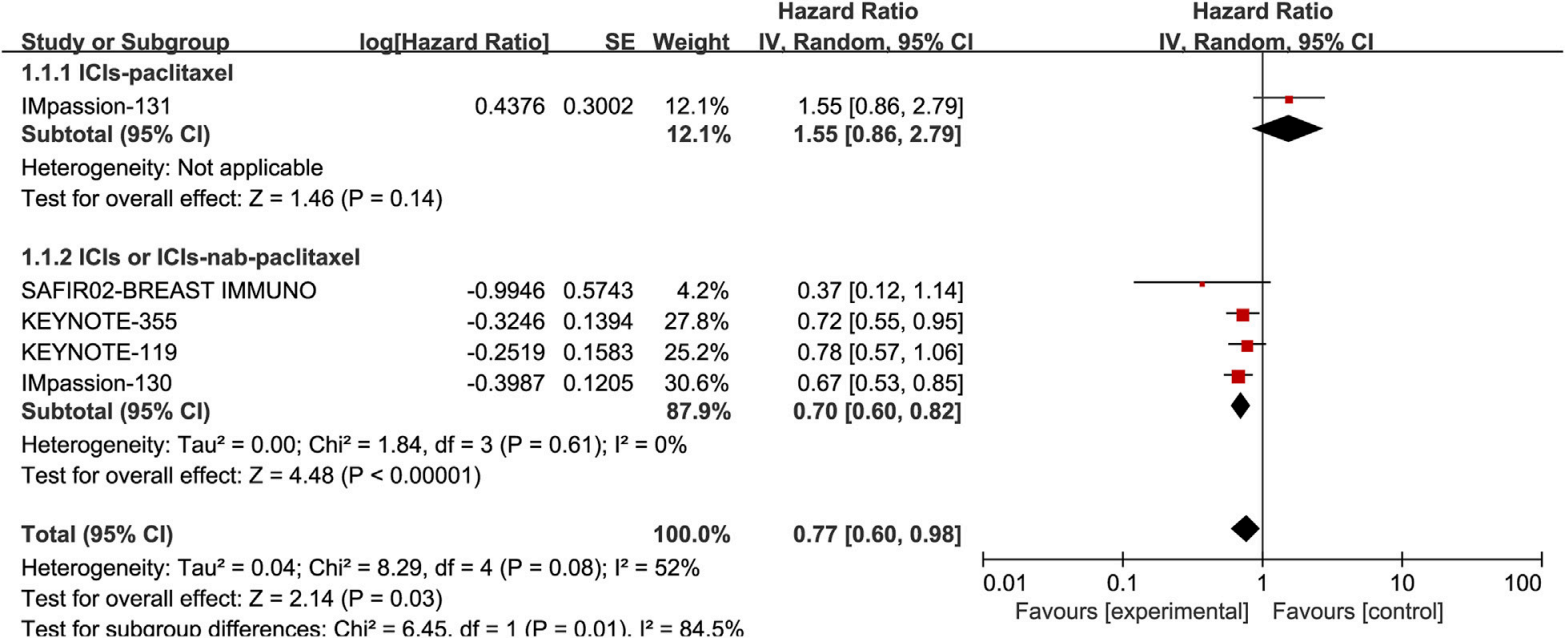
Subgroup analysis of the effect of ICIs combined with paclitaxel or not on OS in PD-L1 positive TNBC patients.

Five studies, including 3105 patients, reported the information of OS with a total of 1210 PD-L1 positive patients and employed a random-effects model for analysis because of obvious heterogeneity (*I* ^*2*^ = 52%, *p* = 0.08). The benefit of OS was found in ICIs, and ICIs combined with chemotherapy compared to standard chemotherapy (HR: 0.77, 95% CI: 0.60–0.98, *p* = 0.03; Figure 3). The subgroup analysis based on ICIs with or without chemotherapy did not reduce heterogeneity (Figure 5). Notably, the results of the subgroup analysis showed that patients had decreased OS when ICIs were combined with solvent-based paclitaxel (Figure 6).

Two studies comprising 1349 PD-L1 negative patients provided the data of OS. We used a random-effects model for analysis due to potential heterogeneity (*I* ^*2*^ = 50%, *p* = 0.16). The result revealed no significant OS benefit in patients who received ICIs therapy (HR: 0.84, 95% CI: 0.44–1.58, *p* = 0.59).

### Sensitivity analysis

**FIGURE 7:**
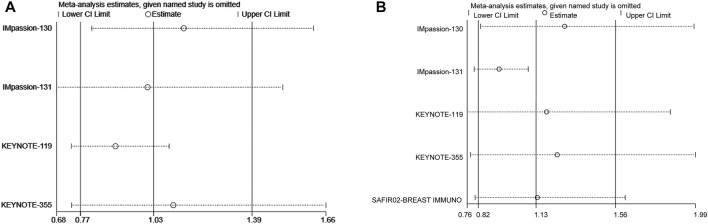
Sensitivity analysis **(A)** for progression-free survival and **(B)** for overall survival.

Sensitivity analysis was performed by excluding each study one by one to determine the reliability of the results. The results showed stable pooled HRs (Figure 7).

Publication Bias.

**FIGURE 8:**
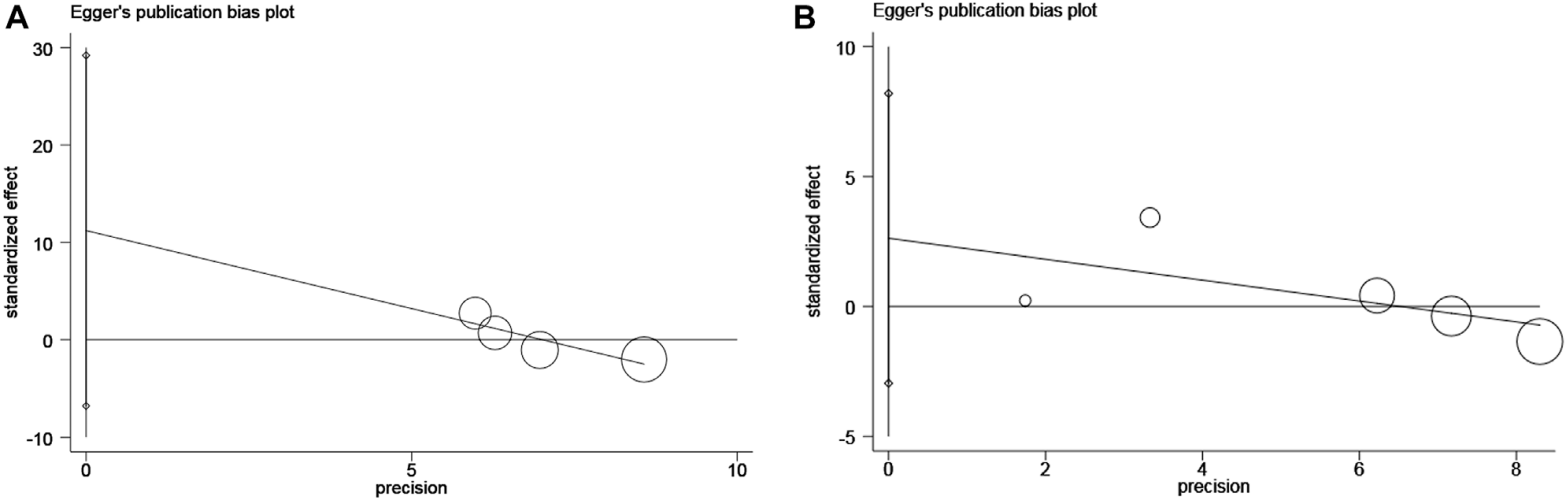
Egger’s test of publication bias **(A)** for progression-free survival and **(B)** for overall survival.

The publication bias of the included studies was assessed by Egger’s test, and the results showed that there was no publication bias (PFS: *p* = 0.116, OS: *p* = 0.231; Figure 8).

## Discussion

TNBC has become the mainstay in malignant tumor immunotherapy due to its high tumor mutational burden, increased infiltration of immune cells and high expression of PD-L1. Currently, the PD-1/PD-L1 pathway is the most prominen target of immunotherapy in locally advanced or metastatic TNBC (Lipson et al., 2015; Hartkopf et al., 2016; Pusztai et al., 2016). IMpassion130 was the first phase III clinical trial to demonstrate the efficacy of first-line immunotherapy in advanced TNBC, atezolizumab plus nab-paclitaxel improved both PFS and OS compared with placebo plus nab-paclitaxel in the PD-L1 positive TNBC. However, the subsequent study of IMpassion131 found that atezolizumab combined with paclitaxel had no benefit for PD-L1 positive metastatic TNBC. KEYNOTE-355 study explored the clinical efficacy of pembrolizumab plus chemotherapy *versus* placebo plus chemotherapy in the first-line treatment of metastatic TNBC, preliminary results confirmed that pembrolizumab combined with chemotherapy significantly improved PFS compared with placebo combined with chemotherapy for TNBC patients with a combined positive score ≥10. It is worth thinking deeply about these inconsistent findings. Therefore, searching for reliable biomarkers for ICI therapy to screen out the optimal beneficiary population is an important clinical problem that needs to be solved.

In terms of PFS, the effect of ICIs combination chemotherapy in first-line was better than that with ICIs alone in post-line. The main reasons for this result might be as follows: first, due to delayed onset and the pseudoprogress in immunotherapy (Wang et al., 2018; Failing et al., 2019), chemotherapy could reduce tumor burden in patients in the shortest time and cause the declination of tumor interstitial fluid pressure (TIFP). The difficulty in monoclonal antibodies’ entrance into the tumor is due to an increased TIFP. Consequently, the decrease of TIFP could promote the entry of macromolecular substances such as monoclonal antibodies into the tumor, thereby improving the effect of antitumor immunotherapy. Meanwhile, the hypoxic state of tumor microenvironment was improved by reducing TIFP, and then alleviated the immunosuppression of T-cells (Hofmann et al., 2013; Baronzio et al., 2015; Patel et al., 2021). Second, factors leading to immunosuppression include: PD-1/PD-L1, cytotoxic T-lymphocyte-associated antigen 4 (CTLA-4), lymphocyte activation gene 3 (LAG-3), and overexpression of androgen receptor (Cimino-Mathews et al., 2015; Andrews et al., 2017; Pardoll et al., 2021; Guan et al., 2022). These factors made it impossible to fully activate T-cells activity by the inhibition of only the PD-1/PD-L1 signal pathway and led to insignificant effects of ICIs alone. Thus, a synergistic anti-tumor effect can result from the combination of PD-1/PD-L1 inhibitors and other drugs which could affect the activity of immune, including 1) Chemotherapy drugs. Chemotherapy drugs could directly and indirectly enhance immune activity. Paclitaxel and gemcitabine could enhance antitumor immune responses by eliminating immunosuppressive cells (myeloid-derived suppressor cells, regulatory T cells) and promoting the activation of immune cells such as the maturation of natural killer cells and the activation of cytotoxic lymphocytes. Chemotherapy could fully expose tumor antigens by killing tumor cells and improve anti-tumor immune recognition, which provide a good antigenic basis for the application of ICIs, thus improving the efficacy of immunotherapy (Lesterhuis et al., 2011; Roselli et al., 2013; Bracci et al., 2014; Wang et al., 2017). 2) Costimulatory molecule. 3) Androgen receptor antagonist. In addition, the lines of treatment might also be one of the factors affecting the efficiency of immunotherapy (first-line in the ICIs combination chemotherapy group *versus* post-line in the ICIs alone). The increased tumor burden and the decreased autoimmune potential of patients with post-line therapy were also a aspect that should be considered.

In terms of OS, with similarities to various baseline characteristics (age, sex, race, tumor stage, the status of PD-L1) among included clinical studies, it was speculated that paclitaxel might be the most likely source of heterogeneity given that the treatment regimen of this study were ICIs combined with paclitaxel, while the other three studies were either ICIs alone or ICIs combined with nab-paclitaxel. The subgroup analyses demonstrated significant OS benefit in PD-L1 positive TNBC with immunotherapy but without paclitaxel. The reasons for the absence of clinical benefits of atezolizumab combined with paclitaxel compared with atezolizumab combined nab-paclitaxel were as shown below: Different chemotherapy drugs for immune combination led to different synergistic effects. Because paclitaxel is highly lipophilic and insoluble in water, the vehicle of paclitaxel was polyoxyethylene castor oil and absolute ethanol, the vehicle system could easily cause severe allergic reactions. Therefore, glucocorticoids pretreatment is clinically required to prevent allergic reactions before administration. However the efficacy of immunotherapy might be impaired due to glucocorticoids pretreatment with paclitaxel. In the IMpassion131 trial, the effect of weekly high-dose glucocorticoids on the efficacy of immunotherapy was an important factor. Additionally, paclitaxel had high systemic toxicity due to the lack of specific distribution in tumor tissue, leading to poor tolerance after long-term application. Albumin-bound paclitaxel belonged to a nano-drug delivery system, which greatly improved the solubility of the drug and avoided the use of polyoxyethylene castor oil, thus the occurrence of allergic reactions was greatly reduced (Kundranda et al., 2015). Moreover, albumin-bound paclitaxel could passively target tumor tissue by utilizing the enhanced permeability and retention (EPR) effect of blood vessels of tumor tissue (Kouchakzadeh et al., 2015), thereby reducing systemic toxicity, improving patient tolerance and the effect of anti-tumor therapy. The differences in the dosage forms of paclitaxel and nab-paclitaxel might result in different therapeutic effects of the two drugs in combination with immunotherapy drugs, which were able to provide some guidance for the choice of clinical chemotherapy regimens.

Finally, the limitations of this study include; 1) Different antibodies, cutoffs, cell types and scoring criteria were used to assess PD-L1 expression. PD-L1 expression was assessed by IHC using SP142 antibody for atezolizumab (IC ≥ 1%), 22C3 antibody for pembrolizumab (CPS≥10%), and SP142 antibody for durvalumab (IC ≥ 1%). In the exploratory analysis of IMpassion130 study, SP142 antibody was used to detect the PD-L1 expression by IHC in advanced TNBC on tumor-infiltrating immune cells, and the IC positive population of PD-L1 accounted for 41% in TNBC, which included most tumor cell PD-L1 positive patients (8.7%). IC-positive population could achieve clinical benefit regardless of the expression of PD-L1 on tumor cell. Therefore, PD-L1 expression on IC was a biomarker for predicting the benefit of atezolizumab combined with nab-paclitaxel in metastatic TNBC. Besides, the exploratory analysis also assessed the conformity of 22C3 and SP142 in detecting PD-L1 expression, about 80% concordance was observed between IC ≥ 1% (SP142) and CPS≥10% (22C3) (Rugo et al., 2020; Schmid et al., 2020). However, it should be noted that these two approaches were not equivalent (Dill et al., 2017; Torlakovic et al., 2020; Noske et al., 2021). 2) Only five RCTs were included in our meta-analysis, and we could not performed a detailed subgroup analysis due to the small number of included studies, which might lead to bias. In addition, some clinical RCTs in TNBC patients are currently ongoing, and further analysis of clinical data was required to draw sound conclusions in the future. 3) The differences between PD-1 and PD-L1 inhibitors were also worth considering.

## Conclusion

In our meta-analysis, we suggested OS benefit in PD-L1 positive advanced or metastatic TNBC, and the subgroup analysis showed that ICIs combination nab-paclitaxel or ICIs alone might be a better choice compared with ICIs combination paclitaxel in the PD-L1 positive TNBC group. In terms of PFS, no significant PFS benefit was found in PD-L1 positive patients, but the subgroup analysis indicated that a significant benefit of PFS was observed for ICIs combination chemotherapy compared to ICIs alone in the first-line treatment in PD-L1 positive TNBC. No significant improvement was observed for OS or PFS in PD-L1 negative group. The results of this meta-analysis may be beneficial to clinicians in forming better treatment strategies to manage TNBC patients. However, further research is needed, given the limited number of studies currently available for data analysis.

## Data Availability

The original contributions presented in the study are included in the article/supplementary material, further inquiries can be directed to the corresponding author.
